# Pet Reptiles in Poland as a Potential Source of Transmission of *Salmonella*

**DOI:** 10.3390/pathogens11101125

**Published:** 2022-09-29

**Authors:** Marta Dec, Magdalena Zając, Andrzej Puchalski, Klaudiusz Szczepaniak, Renata Urban-Chmiel

**Affiliations:** 1 Department of Veterinary Prevention and Avian Diseases, Faculty of Veterinary Medicine, University of Life Sciences in Lublin, 20-033 Lublin, Poland; 2 Department of Microbiology, National Veterinary Research Institute, 24-100 Puławy, Poland; 3 Department of Parasitology and Fish Diseases, Faculty of Veterinary Medicine, University of Life Sciences in Lublin, 20-033 Lublin, Poland

**Keywords:** *Salmonella*, reptiles, zoonosis, serotypes, antimicrobial susceptibility

## Abstract

Reptiles are considered a potential source of *Salmonella* transmission to humans. The aim of this research was to determine the incidence of *Salmonella* in pet reptiles in Poland and to examine *Salmonella* isolates with regard to their biochemical characteristics, serotype, antimicrobial susceptibility, and pathogenic and zoonotic potential. The research material consisted of 67 reptile faeces samples. The taxonomic affiliation of the *Salmonella* isolates was determined by MALDI-TOF mass spectrometry, biochemical analyses, and serotyping; whole genome sequencing (WGS) analysis was performed on three isolates whose serotype could not be determined by agglutination. The antimicrobial susceptibility of the *Salmonella* isolates was determined by the broth dilution method, and in the case of some antimicrobials by the disk diffusion method. The pathogenic and zoonotic potential of the identified serotypes was estimated based on available reports and case studies. The presence of *Salmonella* was confirmed in 71.6% of faecal samples, with the highest incidence (87.1%) recorded for snakes, followed by lizards (77.8%) and turtles (38.9%). All isolates (n = 51) belonged to the species *S. enterica*, predominantly to subspecies I (66.7%) and IIIb (25.5%). Among these, 25 serotypes were identified, including 10 that had previously been confirmed to cause reptile-associated salmonellosis (RAS). *Salmonella* isolates were susceptible to all antimicrobial substances used except streptomycin, to which 9.8% of the strains showed resistance. None of the strains contained corresponding resistance genes. The study demonstrates that pet reptiles kept in Poland are a significant reservoir of *Salmonella* and contribute to knowledge of the characteristics of reptilian *Salmonella* strains. Due to the risk of salmonellosis, contact with these animals requires special hygiene rules.

## 1. Introduction

Salmonellosis is the second most frequently reported zoonotic disease in the EU, after campylobacteriosis. In 2020, 52,702 cases (13.7 per 100,000 population) were reported in the EU. Nearly 30% of patients required hospitalization, and the mortality rate was 0.19% [1]. In the United States, approximately 1.35 million human infections, 26,500 hospitalizations, and 420 deaths occur every year [2]. In Poland, the number of salmonellosis cases has remained stable in the last few years, ranging from about 9000 to 10,000 cases per year (26 per 100,000 in 2017). The disease is noted mainly in children aged 0–4 (~34%) and 5–9 years (~19%) and in the elderly 60+ (~17%). In 2017, hospitalization was required for over 60% of patients, and 10 patients died [3,4]. Food poisoning (97.1% of cases) concerned mainly children, while extraintestinal forms (2.9%) most often concerned people aged over 60. The most frequently isolated serotype was *S.* Enteritidis (75.5%) [3].

The genus *Salmonella* includes two species, *S. enterica* and *S. bongori*, with *S. enterica* further subdivided into six subspecies: *enterica* (I), *salamae* (II), *arizonae* (IIIa), *diarizonae* (IIIb), *houtenae* (IV), and *indica* (VI). According to the last published supplement (no. 48–2014) of the White–Kauffmann–Le Minor scheme, 2659 *Salmonella* serovars had been identified [5]. The vast majority of human salmonellosis is caused by strains of *S. enterica* subsp. *enterica*, within which as many as 1586 serotypes are distinguished [6].

Although the most common source of *Salmonella* infection in humans is contaminated food of animal origin, such as eggs and meat, about 6% of salmonellosis cases (11% of infections in persons <21 years old) are acquired through direct or indirect contact with reptiles [7]. These animals are well-known asymptomatic carriers of a wide variety of *Salmonella* serovars, which can cause non-typhoidal salmonellosis (NTS) in humans. Despite many reports of the presence of *Salmonella* in reptiles, awareness of the dangers of exposure to these animals is still limited. Reptiles are popular as pets in many countries. Across Europe, the population of captive reptiles is over 9 million, with the most living in the United Kingdom (1,450,000), Italy (1,365,000), Spain (~1,240,000), and France (1,090,000). The estimated number of captive reptiles in Polish households in 2020 was 215,000 [8]; however, the frequency of RAS in Poland is not known as there is no obligation to conduct an epidemiological interview with patients. In addition to *Salmonella* transmission via direct human–animal contact, indirect transmission may also play an important role in human infections. Reptiles are not always kept in terrariums; they can often move freely indoors and come into contact with furniture and other objects, thus contaminating the owner’s environment. People can also come into contact with reptiles at reptile exhibitions or at resorts, where street traders encourage tourists to pick up and photograph themselves with snakes or other exotic reptiles [9]. Reptile-related salmonellosis (RAS) mainly affects infants and young children, often resulting in self-limiting 4–7-day gastroenteritis, known as non-typhoidal non-invasive salmonellosis. In some cases, however, *Salmonella*, which normally causes diarrhoea, enters the bloodstream and spreads through the body, leading to sepsis, septic arthritis, meningitis, myocarditis, and even death. Such invasive non-typhoidal *Salmonella* infections are most common in persons in high-risk groups, e.g., young children, the immunocompromised, and the elderly, and they require antibiotic therapy [6,10,11].

Given the growing interest in reptile breeding and the associated potential risk of salmonellosis in humans, the aim of our research was to determine the prevalence of *Salmonella* in pet reptiles in Poland and to examine *Salmonella* isolates for their biochemical characteristics, serotype, and antimicrobial susceptibility. The identification of reservoirs of human-threatening microbes, such as *Salmonella*, is important for assessing the risks associated with their spread and potential infection. An additional aim was to use literature data to estimate the pathogenic and zoonotic potential of the *Salmonella* serotypes identified in this study.

## 2. Materials and Methods

### 2.1. Collection of Faecal Samples

The research material consisted of faecal samples collected between 2017 and 2020 from 67 reptiles kept as pets: snakes (n = 31), lizards (n = 18), and tortoises (n = 18). The animals did not show any symptoms of disease and came from private owners (n = 53) and pet shops (n = 14) in Lubelskie Province, Poland. One or two samples were taken from households, and seven samples from different terrariums were taken from each of the two pet shops. The stool samples were collected in sterile cups or collected with a swab while cleaning terrariums.

### 2.2. Salmonella sp. Isolation

Stool specimens collected with a swab were suspended in peptone water (Buffered Peptone Water, Oxoid, UK) and incubated for approximately 18 h; then, 1 mL of inoculum was transferred into 9 mL of Rappaport Vassiliadis Soya (RSV) Peptone Broth (BTL, PL) and incubated at 41.5 °C for 24 h under aerobic conditions according to the PN-EN ISO 6579:2003/A1:2007 standard. One loopful (10 µL) of the incubated broth was streaked onto each of the triple plates: xylose–lysine–deoxycholate (XLD) agar (Oxoid, UK), Salmonella–Shigella (SS) agar (Oxoid, UK), and Brilliant Green Agar (BGA) (Oxoid, UK). Single colonies (1 or 2 from each plate) with typical *Salmonella* morphology were inoculated on TSB (Trypticase soy broth; Oxoid Ltd., Oxoid, UK), and pure cultures supplemented with 20% glycerol were stored at −80 °C for further analysis.

### 2.3. Identification of Salmonella by MALDI-TOF Mass Spectrometry

The collected isolates grown on TSB agar were identified by matrix-assisted laser desorption ionization-time of flight (MALDI-TOF) mass spectrometry (MS) using a standard ethanol/formic acid extraction method [12]. The mass spectra obtained from each isolate were processed with the MALDI Biotyper 3.1 software package (Bruker, Germany), and the results were shown as the top 10 identification matches along with confidence scores ranging from 0.000 to 3.000, according to the manufacturer’s criteria (www.bruker.com accessed on 1 March 2022).

### 2.4. Biochemical Profiles of Salmonella Strains

In order to assign *Salmonella* isolates to species and subspecies, the API 20E test (Biomerieux, PL) and the series of biochemical tests listed in the White-Kauffmann-Le Minor scheme were performed [13]. The ability to produce β-glucuronidase was determined by culturing the isolates on Tryptone Bile X-Glucuronide (TBX, BTL, PL) agar. Utilization of o-nitrophenyl-6-D-galactopyranoside (ONPG) and sorbitol was assessed using the API 20E test (bioMerieux, PL). Ewing’s malonate modified broth (Biomaxima, PL) was used to evaluate the ability of the strains to utilize sodium malonate. The utilization of galacturonate and mucate was determined using homemade broths [14]. To assess the ability of *Salmonella* strains to utilize dulcitol, lactose and salicin, the isolates were inoculated in peptone water containing 1% sugar and a pH indicator (a 2% solution of bromocresol purple was added to the broth in a volume of 100 µL per 5 mL). The results were read after 24 and 48 h of incubation at 36 °C. The change in the colour of the medium from red to yellow or yellow-brown indicated the utilization of carbohydrates.

To determine the ability of *Salmonella* isolates to utilize L(+)-tartrate (dT+ strains), an indirect method was used, consisting of detection of the sequence located in the region between the *STM 3357* and *STM 3356* genes in the genome of the strains. The *STM 3356* gene encodes a protein responsible for the transport of cations in the L(+)-tartrate fermentation pathway. To discriminate between dT- and dT+ strains, multiplex PCR was performed using primers 166, 167, ST11 and ST15 and DreamTaq polymerase (Thermo Fisher Scientific, USA) [15]. ST11 and ST15 enable the detection of DNA regions characteristic of the genus *Salmonella*, and primers 166 and 167 were used to amplify regions associated with L(+)-tartrate utilization.

### 2.5. Serotyping

To determine *Salmonella*, antigenic structure strains were streaked on Nutrient Agar (NA) (Biomaxima, PL) and AKG plates [16] (home-made, PIWET, PL) and incubated at 37 °C for 24 h. Then, isolates were serotyped with specific O- and H-antisera (Si-fin, DE; SSI Diagnostica, DK) by using agglutination slide tests. The presence of granular agglutination after the application of isolate with specific antisera was classified as a positive result. Based on these results, the composition of O- and H- antigens was established, and serovar names were identified according to the White–Kauffmann–Le Minor scheme [13], including the latest supplement (no. 48) [5].

### 2.6. Whole Genome Sequencing (WGS) and Bioinformatics Tools

Whole genome sequencing was performed for the three strains (nos. 33B, 50, and 51) whose serovar could not be determined in the agglutination test, as well as for one strain of *S.* Muenchen (no. 33A). DNA was extracted using the Maxwell^®^ RSC Cultured Cells DNA Kit—Automated DNA Purification from Mammalian and Bacterial Cultured Cells (AS1620 Promega, Madison, WI, USA) according to the manufacturer’s instructions with the Maxwell^®^ RSC Instrument (Promega, Madison, WI, USA). For yield and purity checks, all samples were measured with NanoDropTM One following extraction (Thermo Scientific, Waltham, MA, USA). DNA libraries prepared with the NextSeq MidOutput Kit 2 × 300 (Illumina, Inc., San Diego, CA, USA) according to the manufacturer’s instructions were sequenced with the NextSeq platform (Illumina, Inc., San Diego, CA, USA). The FastQC 0.11.5 tool was used to check the quality of raw sequencing data, and Trimmomatic 0.36 [17] was used to remove the low-quality sequences at the ends of reads. Corrected reads were assembled de novo by SPAdes v.3.15.3 [18]. The SeqSero 1.2 tool was used to identify the *Salmonella* serovars based on curated databases of *Salmonella* serotype determinants (*rfb* gene cluster, *fliC* and *fljB* alleles) [19].

Resistance genes were identified using the ResFinder [20], available at the Center for Genomic Epidemiology web server (https://cge.cbs.dtu.dk/services, accessed on 4 July 2022), and the AMRFinderPlus tool (https://www.ncbi.nlm.nih.gov/pathogens/antimicrobial-resistance/AMRFinder/, accessed on 4 July 2022), with a 90% threshold for identity with the reference and minimum 80% coverage of the gene length. Detection of genes responsible for lactose uptake and utilization was performed in RAST v2.0 (https://rast.nmpdr.org/, accessed on 4 July 2022). The genomic sequences of *Salmonella* strains were deposited in the GenBank database (BioSample/Acc. No. SAMN25211031- SAMN25211033 and SAMN25046248).

### 2.7. Evaluation of Pathogenic and Zoonotic Potential of Identified Salmonella Serovars Based on Review of Cases

To assess the pathogenic and zoonotic potential of the *Salmonella* serotypes identified in this study, a review of literature from PubMed and other online resources was performed. Reports from the Center for Disease Control and Prevention (CDC), European Food Safety Authority (EFSA) and others were searched for information on the frequency of human infections with specific serotypes. A database search to determine zoonotic potential was performed using the search terms “*Salmonella*”, “infection”, “reptile”, and “zoonosis”, in addition to the names of specific serovars.

*Salmonella* serotypes that have been reported to cause illness with a frequency of at least 0.05 cases per 100,000 population annually (which corresponds to >150 cases in the USA and >220 cases in the EU annually) were considered to have high pathogenic potential. All serotypes that have been reported to induce RAS were considered to have high zoonotic potential.

### 2.8. Evaluation of Antimicrobial Susceptibility of Salmonella Isolates

The drug susceptibility of *Salmonella* isolates to 16 antimicrobial substances was determined using the broth dilution method [21] (for ampicillin (AMP), cefotaxime (CTX), ceftazidime (CFZ), meropenem (MEM), nalidixic acid (NA), ciprofloxacin (CIP), chloramphenicol (CHL), gentamicin (CN), trimethoprim (W), sulfamethoxazole (SMX), tetracycline (T), colistin (CT), and azithromycin (AZM)) or the disk diffusion method [22] (for streptomycin (S, 10 µg), amikacin (AK, 30 µg) and nitrofurantoin (F, 100 µg)). Ready-to-use Sensititre EU Surveillance *Salmonella/E. coli* EUVSEC microplates (ThermoFisher Scientific, Waltham, MA, USA) were used for the microdilution method. A bacterial inoculum was prepared by suspending the pure culture grown on NA agar (24 h, 37 °C) in 0.9% NaCl to obtain a density of 0.5 McFarland. A 10 µL volume of the inoculum was added to 11 mL of Muller-Hinton broth (ThermoFisher Scientific, USA). Microplates were inoculated with 50 µL of the bacterial suspension and incubated at 35 °C for 18 h. The *E. coli* ATCC 25922 reference strain was used as a quality control. Strains were considered resistant (non-wild-type, NWT) if the minimum inhibitory concentration (MIC) for a given antimicrobial substance was above the epidemiological cut-off value (ECOFF) established by European Committee on Antimicrobial Susceptibility Testing (EUCAST) [21]. *Salmonella* with MIC values equal or below the ECOFF were recognized as susceptible (wild type, WT; WT ≤ z mg/L, NWT >z mg/L). WHONET software (v.22.7.21) was used for MIC data management (https://whonet.org/, accessed on 25 July 2022). As EUCAST does not indicate epidemiological cut-offs for SMX and CT, the results for these antimicrobials were interpreted based on the clinical cut-offs according to the Clinical and Laboratory Standards Institute (CLSI) [22] (for SMX) and EUCAST [23] (for CT). For S and AK, the strains were classified as susceptible, intermediate or resistant according to CLSI guidelines [22], while EUCAST guidelines [23] were used for nitrofurantoin.

### 2.9. Detection of Aminoglycoside Resistance Genes

All of the *Salmonella* spp. strains that showed phenotypic resistance or intermediate susceptibility to streptomycin were tested for the presence of corresponding resistance genes, i.e., *strA/strB*, *aadA*, *aac(3)-IV* and *aphA1*, by uniplex PCR [24]. Resistance genes were detected using the primers and annealing temperatures shown in Table S1.

### 2.10. Statistical Analysis

The results of the biochemical tests on *Salmonella* isolates were subjected to statistical analysis, which in total included the results of 28 assays, i.e., lactose, dulcitol, salicin, malonate, mucate, galacturonate, β-glucuronidase, L(+)-tartrate and 20 reactions from the API 20E test, including ONPG (Table S2). Positive and negative results were coded as 1 and 0, respectively, in a data matrix (Excel, Microsoft Office 2019), and the unweighted pair group method with arithmetic averages (UPGMA) was used to generate a dendrogram of dissimilarity.

The relationships between the reptile type (snakes, lizards, or turtles) and the presence of *Salmonella* were determined using the chi-square test. Normal distribution of data was tested using the Shapiro–Wilk test. The level of significance was set at *p* < 0.05. All statistical analyses were performed using Statistica 14.0.0 (TIBCO Software Inc., USA).

## 3. Results

### 3.1. Identification and Prevalence of Salmonella Isolates

The identification of putative *Salmonella* isolates (n = 51) grown on XLD, SS or BGA plates was confirmed by MALDI-TOF mass spectrometry. All isolates were identified only up to the *Salmonella* genus. The discrimination power of MALDI-TOF MS was insufficient to determine the species or subspecies of *Salmonella*. For 14 strains (27.45%), the log (score) identification value was in the range of 2.300–3.000, for 32 isolates (62.74%) it was between 2.000 and 2.299, and for 5 strains (9.80%) it was in the range of 1.700–1.999.

The presence of *Salmonella* was confirmed in 48 of 67 faecal samples (71.6%). A total of 35 out of 53 samples from households (66,04%) and 13 out of 14 samples from pet stores (92,85%) were positive. The incidence of *Salmonella* was highest for snakes, i.e., 87.1% (n = 27/31), and slightly lower for lizards, i.e., 77.8% (n = 14/18), while only 38.9% (n = 7/18) of turtle samples contained these bacteria. The occurrence of *Salmonella* in snakes and lizards was significantly more frequent than in turtles (chi^2^ =13.483, df = 2, *p* = 0.001, C = 0.409) (Table 1).

### 3.2. Biochemical Profiles of Salmonella Strains

Detailed results of the biochemical assays of all *Salmonella* isolates as well as subspecies and serotypes corresponding to specific biochemical profiles are presented in the Supplementary Materials (Table S2). Summarized data on the biochemical analysis are presented in Table 2.

The results of the tests with dulcitol, salicine, sorbitol, galacturonate, β-glucuronidase, and malonate were largely consistent with the information on the biochemical characteristics of individual *Salmonella* subspecies provided in the WHO Collaborating Center for Reference and Research on *Salmonella* guidelines [13]. Surprisingly, none of the *Salmonella* isolates tested produced gelatinase. According to the above-mentioned guidelines [13], gelatinase production is characteristic of the subspecies *salamae*, *arizoneae* and *diarizonae*.

Activity of β-galactosidase (ONPG test) was demonstrated in all (n = 13) strains of *S. enterica* subsp. *diarizonae* and *S. enterica* subsp. *arizonae* (n = 2), 1 strain of *S. enterica* subsp. *salamae* (50%) and 10 strains of *S. enterica* subsp. *enterica*, including 8 strains representing the Lattenkamp serotype. The positive results of the ONPG test for *enterica* subspecies strains are surprising, as the vast majority of strains of subsp. I do not produce β-galactosidase and are lactose-negative [13]. Interestingly, 5 of 26 ONPG+ strains did not utilize lactose (Table S2, Table 2).

**Figure 1:**
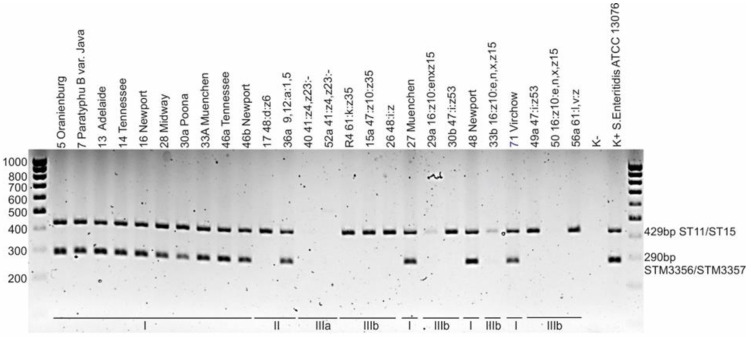
Agarose gel electrophoresis of multiplex PCR employed for discrimination of dT+ and dT- Salmonella strains. The ST11/ST15 amplicon (429 bp) is characteristic of the genus *Salmonella*, and the STM3356/STM3357 amplicon (290 bp) indicates the ability of the strain to utilize sodium potassium tartrate; M—DNA marker, K+—*Salmonella* Enteritidis ATCC 13076. The photo shows profiles of selected *Salmonella* strains.

L(+)-tartrate utilization sequences located in the region between the *STM 3357* and *STM 3356* genes were detected in all *S. enterica* subsp. *enterica* strains, as well as in one (50%) *salamae* strain (No. 36a) (Table 2 and Table S2, Figure 1).

Based on the results of the biochemical tests, and in the case of several strains additionally by serotyping, all isolates (n = 51) were assigned to the species *Salmonella enterica* and to the following subspecies: *enterica* (66.7%, n = 34), *diarizonae* (25.5%, n = 13), *salamae* (3.9%, n = 2), and *arizonae* (3.9%, n = 2) (Table S1). The greatest variation in terms of *Salmonella* subspecies was noted in snakes; among 29 isolates derived from this type of reptile, 15 (52%) belonged to subspecies I, 11 (38%) to IIIb, 2 (7%) to IIIa, and one (3%) to subspecies II. Among 15 isolates isolated from lizards, as many as 13 (86.7%) belonged to subspecies I, and two (13.3%) represented subspecies IIIb. In strains from turtles (total n = 7), subspecies I was most often identified (n = 6; 86%); only one isolate (14%) belonged to subspecies II. The vast majority of isolates of the *diarizonae* (IIIb) subspecies were derived from snakes (n = 11; 85%), and only two isolates (15%) from lizards (Table S2).

**Figure 2:**
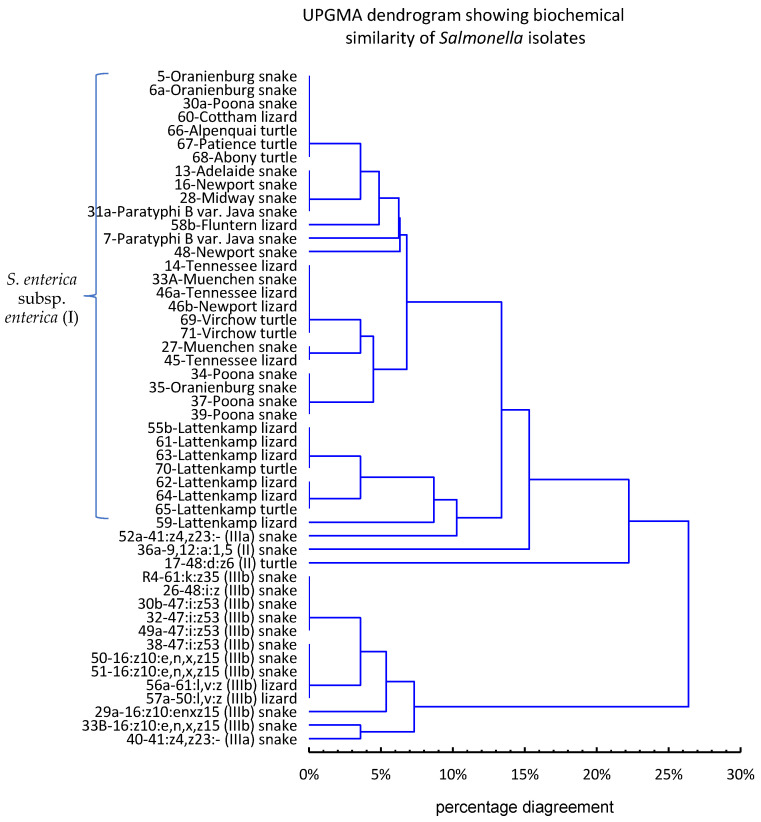
UPGMA dendrogram showing the biochemical similarity of *Salmonella* isolates based on the results of 28 assays (detailed results of individual biochemical tests are presented in Table S1).

The similarity between *Salmonella* isolates based on their biochemical profiles, obtained from the results of 28 biochemical assays, is shown in the dendrogram derived from UPGMA cluster analysis (Figure 2).

At the level of 23% dissimilarity, the strains formed two main clusters—one comprised all *S. enterica* subsp. I and *S. enterica* subsp. II strains and one strain of *S. enterica* subsp. IIIa, while the other comprised *S. enterica* subsp. IIIb strains and one strain of *S. enterica* subsp. IIIa. This analysis showed that the biochemical tests used in the study are helpful in establishing the taxonomic identity of *Salmonella* strains, but they do not always allow for the unambiguous assignment of a strain to a subspecies.

### 3.3. Serotyping

Forty-eight of 51 *Salmonella* isolates were successfully serotyped by the agglutination test. The serotypic pattern of the three other isolates, designated 33B, 50, and 51, was specified as *S. enterica* subsp. *diarizonae* 16:?:? (33B) or *S. enterica* subsp. *diarizonae* 16:z_10_:- (50, 51). Finally, the serotype of these strains was determined to be *S. enterica* subsp. *diarizonae* 16:z_10_:e,n,x,z_15_ based on whole genome sequence analysis. Ultimately, 25 serotypes were identified among 51 *Salmonella* isolates, and all of them were non-typhoidal. *S.* Lattenkamp was the most frequently detected (15.7% of isolates, n = 8), followed by *S.* Poona (7.8%, n = 4), *S. enterica* subsp. *diarizonae* 47:i:z_53_ (7.8%, n = 4), *S. enterica* subsp. *diarizonae* 16:z_10_:e,n,x,z_15_ (7.8%, n = 4), *S.* Newport (5.9%, n = 3), *S.* Oranienburg (5.9%, n = 3), *S.* Tennessee (5.9%, n = 3), *S. enterica* subsp. *arizonae* 41:z_4_,z_23_:- (3.9%, n = 2), *S.* Paratyphi B var. Java (3.9%, n = 2), and *S.* Virchow (3.9%, n = 2). The other 14 serovars were found in single isolates (Table 3).

Some serotypes were found only in snakes, e.g., Oranienburg (n = 3), Poona (n = 4), 16:z_10_:e,n,x,z_15_ (n = 4), and 47:i:z_53_ (n = 4); *S.* Tennessee (n = 3) was characteristic of lizards, and *S.* Virchow (n = 2) was found only in turtles.

### 3.4. Antimicrobial Testing

As many as 90.2% of *Salmonella* isolates (n = 46/51) were sensitive to all antimicrobial substances used in the study (ampicillin, cefotaxime, ceftazidime, meropenem, nalidixic acid, ciprofloxacin, chloramphenicol, gentamicin, trimethoprim, sulfamethoxazole, tetracycline, colistin, nitrofurantoin, streptomycin, amikacin, and nitrofurantoin). Only 5 isolates (9.8%) were resistant to streptomycin, and 52.9% (n = 27/51) showed intermediate susceptibility to this antibiotic. Four of five streptomycin-resistant strains belonged to different serovars of *Salmonella enterica* subsp. *diarizonae*; the other was *S.* Paratyphi B v. Java (Table S3).

### 3.5. WGS

The total length of the final assemblies of four *S. enterica* strains ranged from 5,376,408 bp to 5,984,163 bp. The three strains, whose serotype could not be determined by agglutination (nos. 33B, 50, and 51), were identified as *S. enterica* subsp. *diarizonae* 16:z_10_:e,n,x,z_15_ (Table 4).

The presence/absence of the *lacZ* gene encoding β-galactosidase was correlated with the results of the ONPG test for the four analysed strains. Interestingly, despite the presence of the gene encoding the MFS oligosaccharide transporter, strain 33B did not ferment lactose within 48 h of incubation. Comparative analysis of the lactose operon sequence of strains 33A, 50 and 51 revealed the presence of a non-synonymous mutation (substitution CCG→CTG at nt position 290) in the gene encoding MFS transporter in strain 33B, leading to a Pro97→Leu change.

The *aac(6′)-Iaa* gene, coding for chromosomal-encoded aminoglycoside acetyltransferase, and the *kdeA*, *mdtM*, *emrD* and *acrf* genes, encoding multidrug efflux transporters, were detected in all strains (Table 4). However, the presence of these genes did not correlate with the phenotypic antimicrobial susceptibility profiles.

### 3.6. Pathogenic and Zoonotic Potential of Salmonella Serovars Identified in Reptiles

Reports of human *Salmonella* infections in the USA [25], the EU [1] and Queensland, Australia [26], confirm the prevalence of salmonellosis caused by 19 (76%) of the 25 serotypes identified in this work in reptiles (Table 5). The most accurate data come from the USA, where in 2006–2016 salmonellosis was most often caused by *S.* Newport (~1.4/100,000), *S.* Oranienburg (~0.23/100,000), *S.* Paratyphi B var. Java (~0.13/100,000) and *S.* Poona (~0.11/100,000). *S.* Tennessee (~0.04/100,000) and *S.* Muenchen (~0.03/100,000) were recorded slightly less frequently. Infections caused by serotypes of the subspecies *arizonae*, *diarizonae* and *salamae* identified in this study were reported sporadically (<14 cases per year, <0.004/100,000). In the EU (2018–2020), only 2 of the 25 serotypes, i.e., *S.* Newport (~0.17/100,000) and Muenchen (~0.05/100,000), were confirmed to have caused infections in humans. In Queensland, over 10 years (2007–2016), infections caused by *S.* Virchow were recorded with high frequency (~5.6/100,000), and significantly fewer cases were caused by *S.* Paratyphi B var. Java, *S.* Newport and *S.* Oranienburg (>0.17/100,000) (Table 5). In summary, on the basis of available reports and the adopted criteria, the serotypes with high pathogenic potential (>0.05 cases/100,000 annually) include Newport, Oranienburg, Paratyphi B var. Java, Poona, Virchow, and Muenchen.

Ten (40%) of the 25 serotypes under consideration have documented zoonotic potential. These are the eight serotypes representing subspecies I, i.e., Abony, Cotham, Muenchen, Newport, Oranienburg, Paratyphi B var. Java and Poona, and Tennessee, as well as one serotype of *S. enterica* subsp. *arizonae*, i.e., 41:z_4_,z_23_:-, and one serotype of *S. enterica* subsp. *diarizonae*, i.e., 47:i:z_53_. The pathogen was most often transmitted as a result of contact with turtles, less often with snakes or lizards. The age of patients with RAS ranged from one month to 87 years, but the median estimated in many reports indicates that children aged 3–10 years were most often affected (Table 5).

## 4. Discussion

### 4.1. Prevalence of Salmonella spp. in Captive Reptiles in Poland

Over the past 20 years, there have been a number of reports showing that reptiles, both in the wild and in captivity, are carriers of *Salmonella* [6]. However, the frequency of occurrence of these zoonotic bacteria is highly varied, ranging from 2.1% [38] to 87.5% [39], depending on the geographic region and type of reptiles analysed [6]. The results of the present study showed that pet reptiles bred in Poland are asymptomatic carriers of *Salmonella enterica* strains (71.64% positive samples), with a prevalence of 87%, 78% and 39% in snakes, lizards and turtles, respectively. These findings are largely consistent with the observations of Zając et al. [9], who showed a very high frequency of *Salmonella* in captive reptiles bred in Poland; it was highest in snakes (92.2%), followed by lizards (83.7%) and turtles (60.0%). Our results are also partially in line with the findings of Geue and Löschner [40] and Nakadai et al. [41], who showed the presence of *Salmonella* in 54.1% and 74.1% of faecal samples, respectively, from caged reptiles reared in Germany and Austria and in Japan. The trend of snakes as the most common carrier of *Salmonella* spp. (69.7–76.0% ± 9.3%), lizards as slightly less common (61–69.0% ± 6.7%) and turtles as the least common carriers (2.6–24.3%) has also been confirmed by several other teams of researchers [40,42,43]. A significantly lower frequency of *S. enterica* than that recorded in this study was reported in captive reptiles in Italy (13.61%) [44], Taiwan (30.9%) [42], Indonesia (10%) [45] and Croatia (13%) [46]. Moreover, the Croatian study showed a much higher *Salmonella* prevalence in lizards (48.4%) than in snakes (8.9%) and chelonians (3.8%). The large variation in the frequency of *Salmonella* prevalence in reptiles in different countries may depend on the type of reptile included in the study and the type of diet associated with it. In carnivorous reptiles, *Salmonella* is more likely to occur, as these bacteria may be found in the meat (mice, rats) that these animals are fed. In addition, antibiotics are commonly used on reptilian farms in some countries; antimicrobial substances can destroy the gut microflora of reptiles, including *Salmonella*.

### 4.2. Subspecies and Serovars of Reptile Salmonella Strains

*Salmonella* strains derived from reptiles are very diverse and represent different subspecies and serotypes [9,40,42]. Our findings are consistent with the findings of some other authors [9,40,43], who in captive reptiles in Poland, Germany, Austria and Spain showed predominance of strains of the *enterica* (I) subspecies (53–66.4%) and a lower prevalence of *Salmonella* strains representing the IIIb (11.2–30.3%), IIIa (2–6%), II (3–14.6%) and IV (2–19.6%) subspecies. A lower frequency (34.6%) of *S. enterica* subsp. *enterica* strains and more frequent identification of *arizonae* (IIIa) strains (23.1%) were recorded in captive reptiles (snakes, lizards, and chelonians) in Croatia [46]. A higher prevalence of subspecies IIIb isolates in snakes compared to lizards and turtles was previously also demonstrated by Zając et al. [9]. On the other hand, Geue and Löschner [40] showed that *Salmonella enterica* subsp. *diarizonae* strains occur with a similar frequency in snakes (27%) and lizards (29%).

The lactose fermentation capacity of Lattenkamp strains noted in the present study is a feature rarely found in *S. enterica* subsp. I, in contrast to strains of subspecies IIIa and IIIb [47]. The lack of correlation in several strains between the presence of β-galactosidase and the capacity to utilize lactose may be the result of mutations in the genes encoding lactose transporter or in the regulatory genes of the lactose operon [48]. More detailed studies are needed to confirm the effect of the Pro97→Leu mutation in the MFS transporter gene on the protein’s ability to transport lactose into *Salmonella* cells.

Five of the 25 serotypes described in this study, i.e., Oranienburg, Tennessee, Fluntern, Muenchen and Newport, belong to the 10 most frequently identified serotypes in *Salmonella* strains derived from reptiles in Poland, while the Abony, Adelaide, Cotham, Lattenkamp, Fluntern, Paratyphi B v. Java, Patience, Poona, 47:z_10_:z_35_ (IIIb), 48:i:z (IIIb), and 41:z_4_,z_23_:- (IIIa) serotypes have been detected less frequently [9]. The relatively high frequency of Lattenkamp strains noted in our research is probably explained by the fact that several samples were collected from the same pet shop (six of eight Lattenkamp strains were obtained from lizards from the same shop). *S.* Midway and *S.* Virchow, *S. enterica* subsp. *arizonae* 61:k:z_35_, and *S. enterica* subsp. *diarizonae* 48:d:z_6_, 16: z_10_:e,n,x,z_15_, and 47:i:z_53_ have been reported in reptiles in other countries [41,43,49,50,51,52,53]. The remaining four serotypes recorded in this study, i.e., *S.* Alpenquai, *S.* II 9,12:a:1,5, *S.* IIIb 50:l,v:z, and *S.* IIIb 61:l,v:z, to the best of our knowledge have not yet been described in *Salmonella* strains from reptiles.

### 4.3. Zoonotic Potential of Identified Salmonella Serovars

All serotypes detected in this study were non-typhoid and belonged to either *S. enterica* subsp. *enterica*, which is responsible for over 99% of human infections, and to subspecies II, IIIa and IIIb, which are much less likely to cause human salmonellosis [6]. The Virchow and Newport serovars identified in the study are among the seven serotypes most frequently causing infections in Poland (in 2016 and 2017) [3], while *S.* Newport and *S.* Muenchen rank among the top 11 serotypes most commonly recorded in humans in the EU [1].

Statistical data on the occurrence of RAS in the EU, including Poland, are not available, as there is no obligation to interview patients regarding possible contact with reptiles. The scale of the problem is therefore unknown, although cases of *Salmonella* infections attributed to direct or indirect contact with captive reptiles have been reported in many European countries (in 2008–2017) [28,30,33,34,35,36,54,55,56,57]. Meletiadis et al. [58] estimated the frequency of RAS in Italy at 3% to 7%, and in the 0–21 age group even up to 11.7%. In the USA, regular reports of reptile-associated salmonellosis were conducted from 1994 to 2002 [37,59,60]. Based on the available reports and case studies, some serotypes found in reptiles in Poland, i.e., *S.* Abony, *S.* Cotham, *S.* Muenchen, *S.* Newport, *S.* Oranienburg, *S.* Paratyphi B var. Java, *S.* Poona, *S. enterica* subsp. *arizonae* 41:z_4_,z_23_:- and *S. enterica* subsp. *diarizone* 47:i:z_53_, can be considered to have high zoonotic potential, causing infections not only in infants and babies (under 2 years old) but also in older children and adults with normal immunity [10,11,27,36,61]. Occasionally, RAS in infants and young children (<1–5 years) caused by some of these serotypes (*S.* Abony, *S.* Cotham, *S.* Muenchen, *S.* Newport, and *S.* Oranienburg) has occurred as invasive infections such as septicaemia, meningitis and colitis [28,29,30].

Many RAS reports point to turtles as the most common source of the pathogen [10,11,27,28,34,35]. However, our research shows that only 38.9% of turtles kept in Poland are *Salmonella* carriers, and in Germany, snakes or lizards are most often identified as transmitters of *Salmonella* causing RAS [36].

The diversity of *Salmonella* strains in terms of pathogenic potential is confirmed not only by the analyses of salmonellosis in humans but also by the results of in vitro studies. McWhorter et al. [62] showed that all considered reptile *Salmonella* strains representing 30 different serotypes (including strains of the subspecies I, II, IIa and IIIb) were invasive into both human intestinal epithelial (Caco2) and mouse macrophage cell lines (J774A.1). One of the most invasive was the Paratyphi B ver. Java serotype, and a strain of another serotype identified in our study, *S.* Adelaide, was moderately invasive. Similar results were obtained by Pasmans et al. [63], who demonstrated that all tested reptile *Salmonella* strains representing 44 serotypes belonging to subspecies I, II, IIIb, and IV were able to invade human intestinal epithelial Caco-2 cells, although isolates belonging to subspecies I invaded the Caco-2 cells to a higher extent than those from the other subspecies. What is more, the human isolates invaded the Caco-2 cells to a lesser degree than their saurian counterparts. These in vitro findings correlate with reports of more hospitalizations and cases of invasive infections among RAS patients than in non-RAS ones [30,64].

### 4.4. Antimicrobial Susceptibility

In the present study, we demonstrated that the majority of *Salmonella* strains were sensitive to all tested antimicrobials, except for single strains resistant to streptomycin. Our findings are largely in line with the results of Chen et al. [42], who reported that the prevalence of streptomycin-intermediate-resistant and resistant *Salmonella* strains in captive reptiles in Taiwan was 23.1% and 14%, respectively. The frequency of resistance to other antimicrobial substances was low, ranging from 0% to 9.2%. A relatively low frequency (32.8%) of drug-resistant *Salmonella* strains in captive reptiles in Poland was also shown by Zając et al. [9]. The prevalence of streptomycin-resistant strains reported by these authors was twice as high (25%) as that recorded in this study, while the percentage of strains showing resistance to other antimicrobial substances ranged from 0.2% to 8.1%. A low frequency (12.8%, n = 34/39) of resistant *Salmonella* strains was also reported in captive reptiles in the Czech Republic [65]. Completely different results regarding the drug susceptibility of reptilian *Salmonella* strains were obtained in Italy and Spain, where the frequency of drug-resistant strains (to at least one antimicrobial substance) ranged from 93.1% to 100% [43,44], and the percentage of multi-drug resistant strains was about 70%. Moreover, the Italian study [44] showed a very high percentage of *Salmonella* isolates with resistance (89.7%) and intermediate susceptibility (79%) to streptomycin; however, in contrast to our results, this was not the only type of resistance in the strains tested. The presence of streptomycin-resistant *Salmonella* strains in reptiles (75%) was also reported by Arnafia et al. [45].

The resistance of *Salmonella* strains to streptomycin noted in this study cannot be explained by the use of antibiotic therapy in reptiles, as streptomycin is not used in treatment of these animals in Poland. It also appears that the reduced susceptibility to streptomycin recorded in the study is not a result of acquired resistance, as none of the strains contained the genes that determine streptomycin resistance in *Salmonella* (*strA*, *strB*, *aadA*, *aac(3)-IVa*, and *aphA1*) [66,67]. For all strains, a small zone of inhibition of growth was recorded around the streptomycin discs, i.e., up to 13.5 mm, while a diameter ≤11 mm indicates resistance. Hence, it appears that this reduced susceptibility of reptilian *Salmonella* strains to streptomycin may have an evolutionary basis. The same trait has previously been observed in reptilian strains of *E. coli* [24]. The lack of correlation between the presence of the *aac(6′)-Iaa* gene encoding chromosomal aminoglycoside N(6′)-acetyltransferase (conferring resistance to tobramycin, kanamycin and amikacin) and the phenotypic susceptibility of the *Salmonella* strains (none of the strains were resistant to amikacin) confirms previous reports about the non-functionality of this gene in *Salmonella* [68]. Common occurrence of the *aac(6′)-Iaa* cryptic gene in *Salmonella enterica* strains, including subsp. *diarizonae*, has previously been confirmed by several authors [69,70].

## 5. Conclusions

The research provided valuable data complementing existing knowledge of the prevalence of *Salmonella* strains in reptiles, as well as knowledge of their serotypes, drug susceptibility, and biochemical characteristics. We showed that the pet reptiles kept in households in Poland are common carriers of *Salmonella* strains, including serotypes that had previously been confirmed to cause reptile-associated salmonellosis in humans. Identification of the *Salmonella* reservoir is important in assessing the risk associated with the spread of this zoonotic agent and potential infection.

Reptiles should always be considered a potential source of *Salmonella* transmission, and special hygiene rules should be followed when handling these animals and cleaning terrariums. It is advisable to wash your hands after each contact with reptiles or objects in their living areas. Reptiles should not move freely around the house, especially in the food preparation area, due to possible environmental contamination. When washing terrariums, use disinfectants and disposable gloves. Due to the increased risk of RAS and hospitalization, children under the age of five, immunocompromised people, and the elderly should not touch reptiles or their environment. There is also a need to educate the public, especially reptile owners, about RAS and related preventive measures. Despite the zoonotic risk, the low level of drug resistance of reptilian *Salmonella* strains gives hope for effective antibiotic therapy in the event of infection.

## Figures and Tables

**Table 1:** Frequency of isolation of *Salmonella* from captive reptiles in Poland.

Animals	Number of Samples	Species (Number of Samples)	Diet Group	Number of *Salmonella* Positive Samples	Number of *Salmonella* Isolates
Snakes	31	*Pantheropsis guttatus* (14) *Python regius* (3) *Boa constrictor* (4) *Lampropeltis triangulum* (4) *Morelia pilota* (1) *Orthrophis teaniurus* (5)	carnivore carnivore carnivore carnivore carnivore carnivore	12 (86%) 3 (100%) 4 (100%) 3 (75%) 1 (100%) 4 (80%)	13 3 4 4 1 4
				Total: 27/31 (87.1%)	Total: 29/51 (56.9%)
Lizards	18	*Pogona vitticeps* (6) *Iguana iguana* (4) *Eublepharis macularius* (3) *Furcifer pardali* (5)	omnivore herbivore carnivore omnivore	6 (100%) 3 (75%) 3 (100%) 2 (40%)	7 3 3 2
				Total: 14/18 (77.8%)	Total: 15/51 (29.4%)
Turtles	18	*Testudo horsfieldii* (8) *Testudo hermanni* (9) *Chelonoidis carbonaria* (1)	herbivore herbivore herbivore	4 (50%) 3 (33%) 0	4 3 0
				Total: 7/18 (38.9%)	Total: 7/51 (13.7%)
Total:	67			48/67 (71.6%)	51 (100%)

**Table 2:** Summarized data on the biochemical analysis of *Salmonella* isolates.

Species	*Salmonella enterica*			
Subspecies Number of Strains	*enterica* n = 34	*salamae* n = 2	*arizonae* n = 2	*diarizonae* n = 13
ONPG (24 h)	10 [29%]	1 [50%]	2 [100%]	13 [100%]
Lactose	8 [23.5%] *	0	1 [50%]	10 [77%]
Salicin	0	0	0	0
Dulcitol	34 [100%]	2 [100%]	0	0
Sorbitol	34 [100%]	1 [50%]	2 [100%]	11 [85%]
L(+)-tartrate **	34 [100%]	1 [50%]	0	0
Mucate	34 [100%]	2 [100%]	1 [50%]	0
Malonate	2 [6%]	2 [100%]	2 [100%]	13 [100%]
Galacturonate	0	2 [100%]	1 [50%]	13 [100%]
β-glucuronidase	11 [32%]	1 [50%]	0	13 [100%]
Gelatinase	0	0	0	0

* Eight strains of *S. enterica* subsp. *enterica* that were able to utilize lactose represented the Lattenkamp serotype. ** The ability of *Salmonella* strains to utilize L(+)-tartrate was determined indirectly by detecting the sequence located in the region between the *STM 3357* and *STM 3356* genes.

**Table 3:** *Salmonella* serovars in captive snakes, lizards, and turtles in Poland.

No.	Serovar	Subsp.	Number of Strains (%)	Snakes n = 31	Lizards n = 18	Turtles n = 18	Host	Strain ID
1	9,12:a:1,5	II	1 (2.0)	1			boa constrictor	36a
2	48:d:z_6_	II	1 (2.0)			1	steppe tortoise	17
3	41:z_4_,z_23_:-	IIIa	2 (3.9)	2			milk snake beauty rat snake	40 52a
4	16:z_10_:e,n,x,z_15_	IIIb	4 (7.8)	4			corn snake milk snake beauty rat snake beauty rat snake	29a 33B 50 51
5	47:i:z_53_	IIIb	4 (7.8)	4			corn snake milk snake corn snake beauty rat snake	30b 32 38 49a
6	47:z_10_:z_35_	IIIb	1 (2.0)	1			ball python	15a
7	48:i:z	IIIb	1 (2.0)	1			boa constrictor	26
8	50:l,v:z	IIIb	1 (2.0)		1		panther chameleon	57a
9	61:k:z_35_	IIIb	1 (2.0)	1			corn snake	R4
10	61:l,v:z	IIIb	1 (2.0)		1		panther chameleon	56a
11	Abony	I	1 (2.0)			1	Greek tortoise	68
12	Adelaide	I	1 (2.0)	1			ball python	13
13	Alpenquai	I	1 (2.0)			1	Greek tortoise	66
14	Cotham	I	1 (2.0)		1		leopard gecko	60
15	Fluntern	I	1 (2.0)		1		leopard gecko	58b
16	Lattenkamp	I	8 (15.7)		7	1	bearded dragon leopard gecko bearded dragon bearded dragon bearded dragon green iguana green iguana steppe tortoise	55b 59 61 62 63 64 65 70
17	Midway	I	1 (2.0)	1			corn snake	28
18	Muenchen	I	2 (3.9)	2			boa constrictor milk snake	27 33A
19	Newport	I	3 (5.9)	2	1		corn snake bearded dragon carpet python	16 46b 48
20	Oranienburg	I	3 (5.9)	3			corn snake corn snake boa constrictor	5 6a 35
21	Paratyphi B var. Java	I	2 (3.9)	2			corn snake corn snake	7 31a
22	Patience	I	1 (2.0)			1	Greek tortoise	67
23	Poona	I	4 (7.8)	4			corn snake ball python corn snake corn snake	30a 34 37 39
24	Tennessee	I	3 (5.9)		3		bearded dragon green iguana bearded dragon	14 45 46a
25	Virchow	I	2 (3.9)			2	steppe tortoise Greek tortoise	69 71
	Total number (%)		51 (100)	29 (57)	15 (29)	7 (14)		

**Table 4:** Results of genomic analysis of selected *Salmonella* isolates.

Isolate ID	50 (S21_0833)	51 (S21_0834)	33B (S21_1654)	33A (S21_0821)
Subspecies	IIIb	IIIb	IIIb	I
Serotype	16:z_10_:e,n,x,z_15_	16:z_10_:e,n,x,z_15_	16:z_10_:e,n,x,z_15_	Muenchen (6,8:d:1,2)
Source	beauty rat snake (*Orthriophis taeniurus*)	beauty rat snake (*Orthriophis taeniurus*)	milk snake (*Lampropeltis triangulum*)	milk snake (*Lampropeltis triangulum*)
Acc. No.	SAMN25211032	SAMN25211031	SAMN25211030	SAMN25046248
Genome size	5921031 bp	5922870 bp	5984163 bp	5376408 bp
Contigs	339	293	245	115
Resistance genes	*aac(6′)-Iaa* *, *kdeA*, *mdtM*, *acrF*, *emrD*	*aac(6′)-Iaa*, *kdeA*, *mdtM*, *acrF*, *emrD*	*aac(6′)-Iaa*, *kdeA*, *mdtM*, *acrF*, *emrD*	*aac(6′)-Iaa*, *kdeA*, *mdtM*, *acrF*, *emrD mdsA*, *mdsB*
Lactose operon genes	*lacZ* **, locus_tag L4A35_20235 (oligosaccharide MFS transporter), *lacA* ***, *lacI* **** (Acc. No. JAKJQB010000022.1)	*lacZ*, locus_tag L4B25_00380 (oligosaccharide MFS transporter), *lacA*, *lacI* (Acc. No. JAKJQC010000001.1)	*lacZ*, locus_tag L4A43_06620 (oligosaccharide MFS transporter), *lacA*, *lacI* (Acc. No. JAKJQD010000004.1)	Not found

* cryptic gene; ** *lacZ* coding for β-galactosidase; *** *lacA* coding for galactoside O-acetyltransferase; **** *lacI* coding for transcriptional regulator.

**Table 5:** Pathogenic and zoonotic potential of *Salmonella* serovars identified in the study.

		Pathogenic Potential			Zoonotic Potential					
Serovar	Subsp.	Number of Infections in US in 2006–2016 [25]	Number of Infections in EU in 2018–2020 [1]	Number of Infections in Queensland in 2007–2016 [26]	Host	Age of Patients with RAS/Median [years]	Number of Infections	Country	Year	Reference
Abony	I	62	– ^a^	–	turtle turtle	4 <1	1 1	Japan Belgium	2007–8 2008	[27] [28]
Adelaide	I	1001	–	–						
Alpenquai	I	–	–	–						
Cotham	I	429	–	–	lizards	<1–79/3	160	USA	2012–14	[29]
Fluntern	I	77	–	–						
Lattenkamp	I	17	–	–						
Midway	I	–	–	–						
Muenchen	I	1216	703	–	reptiles ^b^ turtle	<5 <1–60/10	48 132	UK USA	2015 2009	[30] [10]
Newport	I	47,481	2233	160	reptiles ^b^ turtle turtles	<5 1.25 <1–85/6	48 1 124	UK Chile USA	2015 ? 2012	[30] [31] [10]
Oranienburg	I	8012	–	88	reptiles ^b^ turtle bearded dragon	<5 <5 ?	48 26 1	UK USA Czech Republic	2015 2019 2016	[30] [32] [33]
Paratyphi B var. Java	I	4486	–	355	turtle turtle turtle turtle snake ^b^	<1–10 <1–4 1–87/7 1–75/6 50	8 4 107 132 1	Spain Spain USA USA Finland	2010–11 2009 2007 2011 2005–8	[34] [35] [10] [10] [36]
Patience	I	–	–	–						
Poona	I	3844	–	–	turtle turtle turtle turtle turtle snake iguana	<1 to 82/6 5 <1–84/3.5 <1–83/3 <1–75/5 <1 2	61 1 58 78 40 1 1	USA Japan USA USA USA Germany USA	2015 2007–8 2012 2012 2014 2008 2002	[11] [27] [10] [10] [10] [36] [37]
Tennessee	I	1326	–	–	bearded dragon	<1	1	USA	2000	[37]
Virchow	I	980	–	2804						
9,12:a:1,5	II	2	–	–						
48:d:z_6_	II	10	–	–						
41:z_4_,z_23_:-	IIIa	146	–	–	snake or lizard snake	<1 <1–57	2 3	Germany Belgium	2007–8 2008	[36] [36]
16:z_10_:e,n,x,z_15_	IIIb	21	–	–						
47:i:z_53_	IIIb	–	–	–	snake	25	1	Germany	2006	[36]
47:z_10_:z_35_	IIIb	3	–	–						
48:i:z	IIIb	69	–	–						
50:l,v:z	IIIb	3	–	–						
61:k:z_35_	IIIb	–	–	–						
61:l,v:z	IIIb	–	–	–						

a—the serotype was not identified or listed in the report; cases of infections caused by rarely identified or unknown *Salmonella* serotypes were included in the report as one group. b—probable source of infection, not scientifically proven

## Data Availability

All the data presented in the study are included in the article. Further enquiries can be directed to the corresponding author.

## Supplementary Materials

The following supporting information can be downloaded at: https://www.mdpi.com/article/10.3390/pathogens11101125/s1, Table S1: Sequences of primers used to detect resistance genes; Table S2: Biochemical profiles of *Salmonella* strains; Table S3. *Salmonella* susceptibility test results.
